# Analysis of antibiotics resistant genes in different strains of *Staphylococcus aureus*

**DOI:** 10.6026/97320630014113

**Published:** 2018-03-31

**Authors:** Benson Otarigho, Mofolusho O. Falade

**Affiliations:** 1 Department of Biological Science, Edo University, Iyamho, Edo State, Nigeria; 2 Department of Molecular Microbiology and Immunology, School of Medicine, Oregon Health and Science University, Portland, OR USA; 3 Cellular Parasitology Programme, Cell Biology and Genetics Unit, Department of Zoology, University of Ibadan, Ibadan, Nigeria

**Keywords:** *Staphylococcus aureus*, Resistant Genes, Pathogens, Bacteria

## Abstract

The control of Staphylococcus aureus infection is being hampered by methicillin and other resistant strains. The identification of the unique antibiotic resistant genes from the genomes of various strains of S. aureus is of interest. We analyzed 11 S. aureus genomes sequences for Antibiotics Resistance Genes (ARGs) using CARD 2017 platform. We identified 32 ARGs across 11 S. aureus strains. Tet(38), norB, lmrB, mepA and mepR were present across genomes except for S. aureus strain UTSW MRSA 55. The mepA and mepR were found across 11 different genomes. However, FosB3, vgaALC, mphC and SAT-4 were found in UTSW MRSA 55, S.a. strain ISU935 and S.a. strain FDAARGOS_159. The prevalent mode of mechanism of antibiotics resistant was efflux pump complex or subunit conferring antibiotic resistance as well as protein(s). Analysis of norB, ImrB, norA, ImrB, tet (38), sav1866 and mecA have 12 to 14 TMHs. The results help in the understanding of Staphylococcus aureus pathogenesis in the context of antibiotic resistance.

## Background

*Staphylococcus aureus* is a gram-positive bacterium that naturally inhabits human and other animals skin and mucous membranes [1, 2]. In humans, S. aureus lives symbiotically with other bacterial species and can be beneficial to humans because it enables and expands the memory of T-cells [3, 4]. However, this bacterium can infect other tissues and become an opportunistic pathogen [1, 5]. The pathogenic strains produce virulence factors such as potent protein toxins.

Naturally, S. aureus is susceptible to most known antibiotics [3, 4, 6]. However, there are antibiotic-resistant strains of S. aureus [4, 6]. The resistance genes expressed by these strains are mainly acquired from external sources [7, 8]. This could be either natural or due to human actions mainly by antimicrobial abuse, misuse and lead to chromosomal mutation and antibiotic selection [7, 8]. The emergence and worldwide spread of antibiotic-resistant strains of S. aureus such as methicillin-resistant S. aureus (MRSA) is of health and socio-economic importance [7, 9]. Hence, antibiotic resistant strains are major concern globally [9]. Annually, about 23, 000 people die due to antibiotic-resistant bacterial infections [10, 11]. An estimate of 100 trillion USD losses due to antibiotic resistant is known [10, 12].

One of the challenges confronting the treatment of S. aureus infection is resistance to many commonly used antimicrobial drugs [10, 11]. When S. aureus was first discovered, it was easy to treat using available antibiotics. Some years after the introduction of penicillin in 1940 to combat S. aureus, there were strains of the pathogen that were resistant to these antibiotics. Methicillin was developed and introduced to treat penicillin-resistant S. aureus strains in 1961 [13]. The antibiotics, penicillin and methicillin mode of action is very similar and it involves inhibiting the synthesis of cell wall through the stoppage of peptidoglycan formation by the pathogen and finally lysis of the bacterium. In less than a year after the introduction of methicillin, strains of S. aureus were reported to be methicillin resistant and gradually these strains spread globally [14, 15]. MRSA became a deadlier strain, which has become resistant to most β- lactam antibiotics [14]. It is known that certain genes are involved in the resistance to antibiotic drugs [14, 16, 17], which have been transferred from a different bacterium, S. sciuri. It has been suggested that there may be unknown Antibiotic Resistance Genes (ARGs), which involves S. aureus resistance to antibiotics [4, 18]. The recent sequencing of different S. aureus strains genomes as well as development of bioinformatics tools holds great promise for more efficient and high throughput in the identification and characterization of target genes [4, 19]. The mechanisms by which these genes are involved in resistant to antibiotics could also be deduced. These genes could also provide insight into the pathogenesis and biology of the pathogen [19]. Therefore, the identification of the unique antibiotic resistant genes from the genomes of various S. aureus strains is of interest to deduce its mechanism.

## Methodology

### Genome Retrieval and Identification Analyses

**Figure 1:**
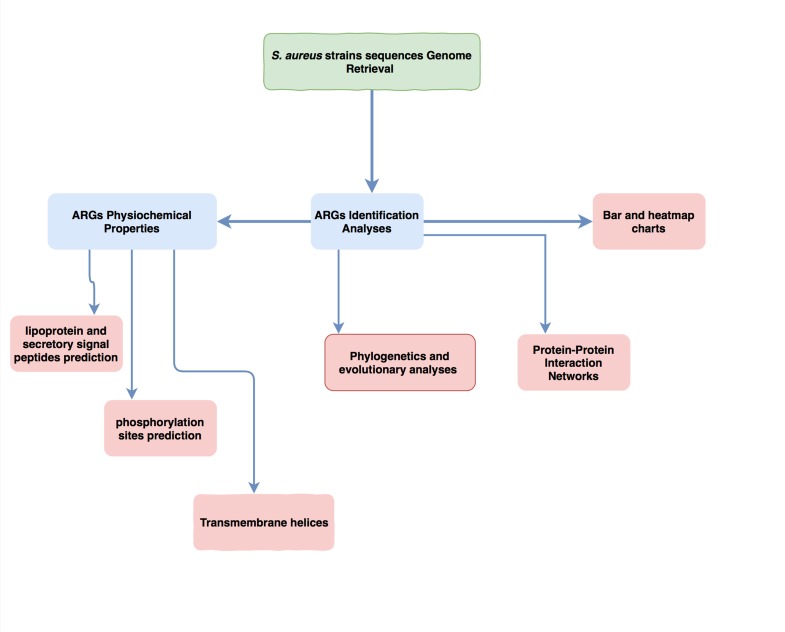
A workflow of the Methodology from S. aureus strain genomes retrieval to various analyses.

The complete genome of S. aureus sequences was downloaded from The National Center for Biotechnology Information (NCBI) Genome Database (https://www.ncbi.nlm.nih.gov/genome). The fasta file format of the genome sequence of 11 strains of bacteria were thoroughly analysed for Antibiotics Resistance Genes (ARGs) on the bulk analysis Resistance Gene Identifier (RGI) or CARD 2017 Platform (https://card.mcmaster.ca/ analyze/rgi) [20]. Default select criteria, which identified gene base on strict or perfect only was used. On the RGI platform each genome sequence file was uploaded and all settings were left at default. To have an inter-relation as well as qualitative and quantitative pattern of these ARGs in the various S. aureus strain, a heatmap chart was constructed using Microsoft Excel 2016 version for Mac. The methodology workflow is presented in Figure 1.

### Physiochemical Properties

Prediction of lipoprotein and secretory signal peptides in grampositive bacteria was done for each sequences using Pred-LiPo (http://bioinformatics.biol.uoa.gr/PRED-LIPO/), a web server that used Hidden Markov Models (HMM). This was validated using CW-Pred (http://bioinformatics.biol.uoa.gr/CW-PRED/), a tool that is also HMM based for the classification of cell wallanchored proteins of Gram-positive bacteria. LipoP (http://www.cbs.dtu.dk/services/LipoP/), SignalP (http://www.cbs.dtu.dk/services/SignalP/) and TargetP (http://www.cbs.dtu.dk/services/TargetP/) (all tools belong to the CBS prediction server) platform were also employed to validate the signaling properties of these proteins and subcellular location of proteins was done. SecretomeP was employed to perform non-classical and leaderless secretion of proteins. Serine and threonine phosphorylation sites in all the obtained antibiotic resistance genes were predicted using NetPhosBac 1.0 platform (http://www.cbs.dtu.dk/services/NetPhosBac/). The Serine and threonine phosphorylation sites were validated using GPS 3.0 Mac version downloaded from (http://gps.biocuckoo.org/online_full.php).

### Phylogenetic analysis

The antibiotic resistance genes in one Fasta file format were edited further using textEdit mac version prior to phylogenetics and evolutionary analyses on Molecular Evolutionary Genetics Analysis (MEGA) platform version 7.0 for Mac, obtained from http://www.megasoftware.net [21]. The 237 sequences were aligned using muscle tools with large alignment (Max iterations = 2) selected while other settings were left at the defaults. Evolutionary history was inferred using the Maximum Likelihood method. The percentage of replicate trees in which the associated taxa clustered together in the bootstrap test (500 replicates) was also analysed. The tree was drawn to scale, with branch lengths in the same units as those of the evolutionary distances used to infer the phylogenetic tree. All positions containing gaps and missing data were eliminated. The phylogenetics and evolutionary analyses were confirmed and validated using Phylogeny.fr platform (http://www.phylogeny.fr) and TreeTop - Phylogenetic Tree Prediction web server Platform (http://www.genebee.msu.su/services/phtree_reduced.html) from the phylogeny tools (http://www.imsc.res.in/~jagan/TOOLS_SEQ_PHYLO.html). The newick format of the tree was exported and opened on FigTree 1.4.2 platform downloaded from http://tree.bio.ed.ac.uk/software/figtree/. The final tree was constructed using the rectangular tree layout. Circular node shape and scale axis were added from the FigTree platform [22].

### Protein-Protein Interaction Networks

Protein-protein interaction network was predicted for each of the ARGs on StringDB Version 10.5 (https://string-db.org) [23]. The sequences for each protein were used in the analyses and S. aureus was selected. The hit with the highest E value and bit score was selected for the final analysis. For each result, molecular action was selected under the setting. Proteins with weak interaction were excluded for further analyses.

## Results

**Figure 2:**
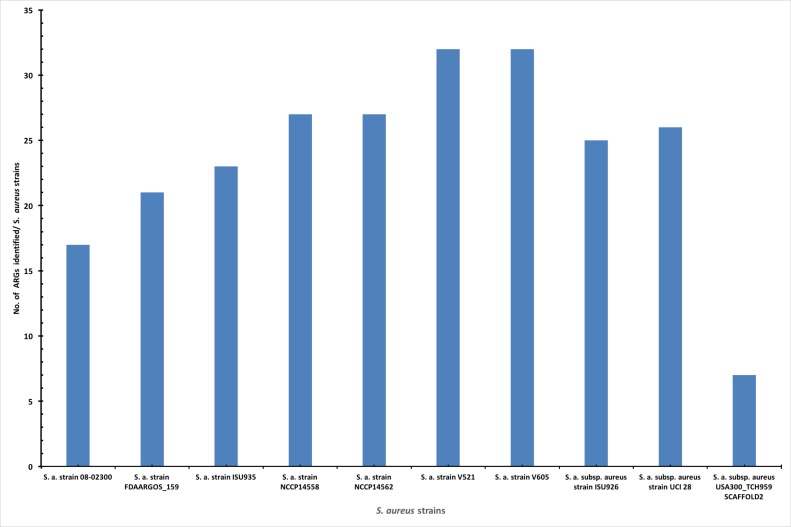
A bar chart showing the different S. aureus strains and the number of the antibiotic resistance genes in each genome. S. aureus V521 and V605 strains had equal number of ARGs; is 32 and that is the highest number among among the 11 genomes studied. While S. a. subsp. aureus USA300_TCH959 SCAFFOLD2 and UTSW MRSA 55 strains have the lowest number with 7 and 8 ARGs.

Genomes of 11 strains of S. aureus that were retrieved from the NCBI are presented in Table 1. Most of the identified ARGs have high expectation (E) value and bit score, with mostly strict cut-off while few were perfect. However, 6 out of the 8 ARGs identified in the S. aureus strain UTSW MRSA 55 were perfect, while the other two were strict. The V521 and USA300_TCH959 SCAFFOLD2 strain of S. aureus with the accession number CP013957.1 and GG697986 had the highest and lowest number of base pairs. After the identification of acquired ARG by various bacteria genomes on the RGI platform, it was noticed that S. aureus strain V521 and V605 had 32 ARGs, which is the highest in the different strains genomes as presented in Figure 2. The S. aureus strain's genome with the lowest ARGs are USA300_TCH959 SCAFFOLD2 and UTSW MRSA 55 that had 7 and 8 respectively.

**Figure 3:**
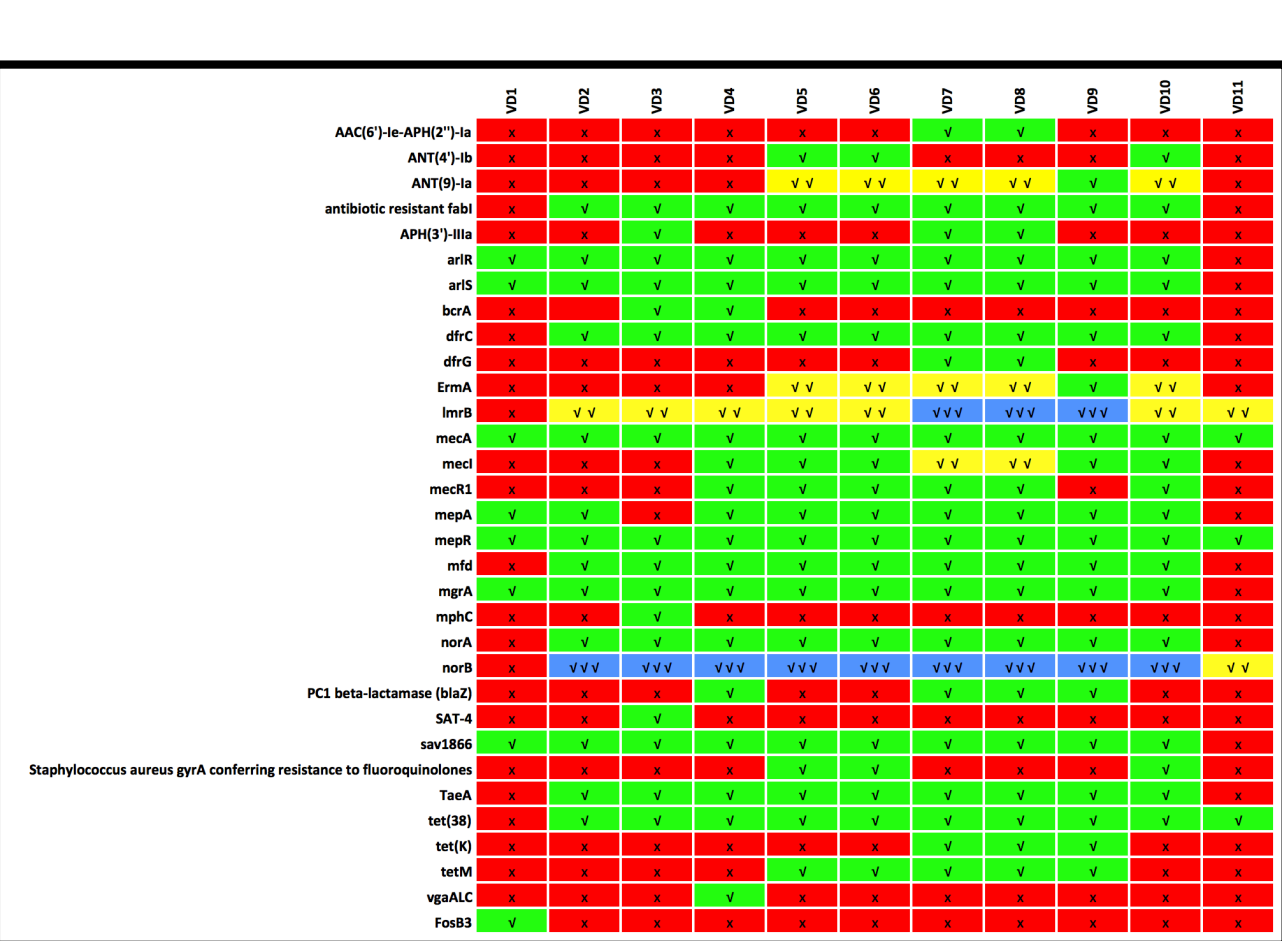
A heatmap that is qualitatively and quantitatively displaying the occurrence of ARGs in the various S. aureus strain genomes is shown. The mepA and mepR are common to all the genome studied while mphC, SAT-4 and vgaALC are unique. The mphC and SAT-4 genes were identified only in S. aureus strain FDAARGOS_159 while the vgaALC and FosB3 was identified only in S. aureus ISU935 and UTSW MRSA 55 strains, respectively

The heatmap chart in Figure 3 presents the relationship of the different S. aureus strains genomes studied in this work and the ARGs identified in each genome. We noticed that two ARGs that include norB and ImrB were identified in triplicate in 9 and 3 S. aureus strain genomes respectively (Figure 3). Hence, the norB gene was the most dominant gene in the different genomes studied. It was also noticed that five ARGs that include AN (9)-la, ErmA, ImrB, mecl and norB were identified in duplicate in 5, 5, 7, 2 and 1 S. aureus strain`genomes respectively (Figure 3). The other ARGs identified were all single in each genome. While mphC, SAT-4 and vgaALC were the least common ARO genes identified across the eleven different S. aureus strains. The mphC and SAT-4 genes were identified only in S. a. strain FDAARGOS_159 while the vgaALC and FosB3 were identified only in S. a. ISU935 and UTSW MRSA 55 strains, respectively (Figure 3 and Table 2).

**Figure 4:**
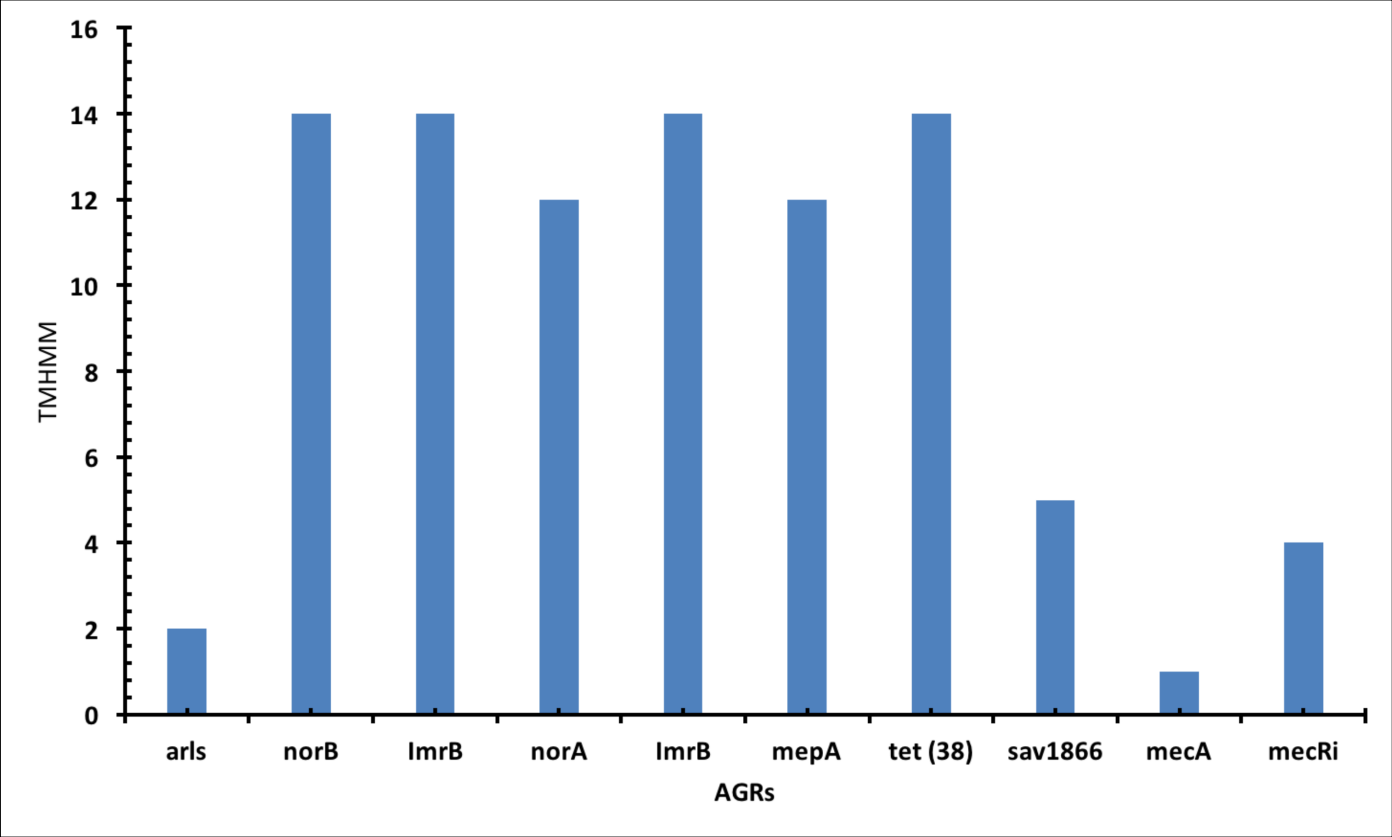
A bar chart shows the antibiotics resistance gene and the number of transmembrane helices. The norB, ImrB and tet (38) had 14 TMHs while norA and mepA had 12 TMHs.

The different novel Antibiotic Resistance Ontology based on the gene names are presented in Figure 3 and 4. All the genomes studied in this work had tet(38), norB, lmrB, mepA and mepR in common except the S aureus strain UTSW MRSA 55. It was noticed also that mepA and mepR were spread across the 11 different genomes. All the ARGs, except for FosB3, identified in the S. aureus strain UTSW MRSA 55 were shared by more than one other strains of S. aureus. Other shared ARGs are presented in Table 2. However, some ARO were unique to just one strain genome. The mphC and SAT-4 genes are unique to S. a. strain FDAARGOS_159 while vgaALC gene is unique to S. a. strain ISU935.

The transmembrane helix (TMH) prediction shows that norB, ImrB, norA, ImrB, tet (38), sav1866 and mecA that have 12 and 14 TMHs (Figure 4). While mecRi, arls and mepA have fewer than 5 TMH. The ARGs are mostly identified as perfect in S. aureus strain UTSW MRSA 55. Almost all the ARGS were identified with protein homolog model. While only antibiotic resistant fabI and S. aureus gyrA conferring resistance to fluoroquinolones were identified with protein variant model. The PC1 beta-lactamase (blaZ) is the only gene that was predicted to have signal peptide cleavage sites in the different identified ARO genes from the various genomes. Localization prediction shows that most of the identified genes in this work are either membrane, cytoplasmic or lipoprotein. We also noticed that most of these genes have more than one phosporylation sites.

The norB, vgaALC, mepA, lmrB and other genes have a single antibiotics resistance mechanism. While mecA, mecR1, arlS, arlR, PC1 beta-lactamase (blaZ), mecI and others have more than one antibiotic resistance mechanisms. The most prevalence mode of mechanism of antibiotic resistance are efflux pump complex or subunit conferring antibiotic resistance as well as protein(s) (norA, norB, sav1866, ImrB, arlB, mgrA, TaeA, tet 38, mepR, arls, vgaALC, bcrA and tet K) and two-component regulatory system modulating antibiotic efflux (mepR, arlR, mgrA and arls). Therefore we noticed that ARGs that have two-component regulatory system modulating antibiotic efflux also carry out the antibiotic resistance are efflux pump complex or subunit conferring antibiotic resistance. Other mechanisms include; antibiotic resistance gene cluster, cassette, or operon [MecA, mecl, mecR1 and PC1 beta-lactamase (blaZ)] and antibiotic inactivation enzyme (ANT(9)-Ia, AAC(6')-Ie-APH(2'')-Ia, SAT-4 and mphC). ARG variant or mutant were also obtained for antibiotic resistant fabI and S. aureus gyrA conferring resistance to fluoroquinolones genes alone. These ARGs that were identified to have variant or mutant were also the ones that possess Single Nucleotide Polymorphisms (SNP); G93A and S85P for antibiotic resistant fabI and gyrA.

Antibiotics targets that were identified include modifying enzyme (ErmA), replacement protein (dfrC, MecA, mecR1, dfrG and mecl) and protective protein (tetM, mfd). The determinant and mechanism as well as antibiotic target and antibiotic molecule of the ARGs were obtained. The antibiotic resistance determinants for beta-lactam (for mecR1, mecA and mecI), aminoglycoside (ANT(9)-Ia, dfrC, AAC(6')-Ie-APH(2'')-Ia and ANT(4')-Ib), lincosamide, macrolide (mphC), streptogramin (for ErmA), diaminopyrimidine (for dfrC), tetracycline (for tetM), fluoroquinolone (for gyrA and mfd), nucleoside antibiotics (SAT- 4), and fosfomycin (for FosB3) as well as isoniazid and triclosan (antibiotic resistant fabI) resistance were also identified across the different ARGs in this study.

**Figure 5:**
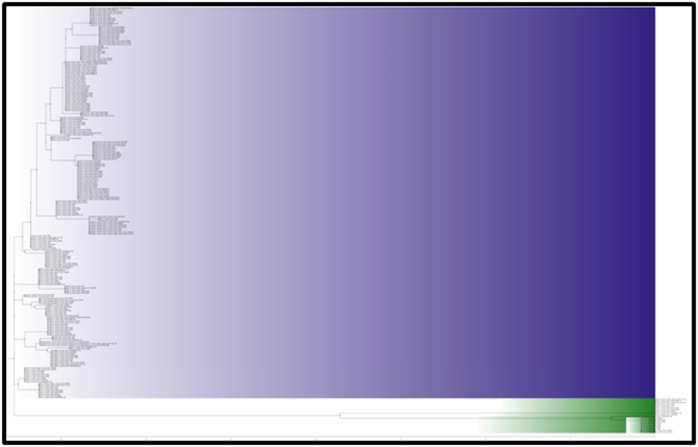
The phylogenetic tree showing the ARGs and how they are related. There are two Major Clades (the major and minor). The Major Clade (purple) contains 29 ARGs while the Minor Claude (green) contain 3 ARGs.

The phylogenetic analysis shows that there are two Major Claudes (the major and minor) as presented in Figure 5. The Major Claude (purple) contained 29 ARGs that have evolved long ago. While the Minor Claude (green) contain 3 ARGs that evolved recently. The ARGs on the Minor Claude are arlR, SAT-4 and mecl, while all others are on the Major Claude. We noticed that the ARGs cluster together based on the gene name in the different strains. All norB, NorA clustered together and same to the other ARGs. However, two exceptions were noticed to this general observation. The first exception was in the unique genes such as mphC, SAT-4, vgaALC and FosB3. The SAT, VgaALC and mphC shared a node with the mecl (on the Minor Claude), mecR1 and mgrA genes respectively, however, the FosB3 distinct itself on the Major Claude. The second exception was for the dfrG (strain V521 and V605) that took a node between the antibiotic resistant fabl for S. aureus 08-02300 strain and the other antibiotic resistant fabl strains on the Major Claude.

**Figure 6:**
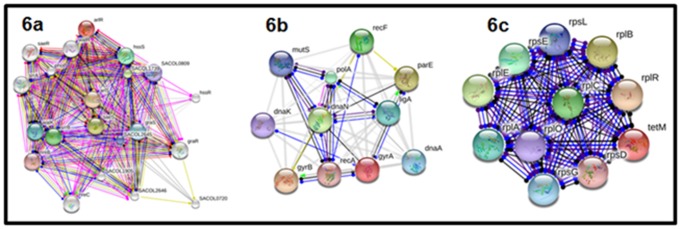
Protein-protein interaction networks for arlS, arlR (6a), gyrA (6b) and Tet (M) (6c) genes. The networks show the reaction and binding of the query genes with a wide range of genes to be involved in antibiotics resistance.

After thorough analyses and extruding proteins with no hit and poor interaction network on the stringDB, the following ARGs; arlR, arlS, gyrA and Tet M were selected for further discussion. The protein-protein interaction networks are presented in Figure 6a-c. The arlS and arlR gene dependently regulate and modulate other genes as presented in Figure 6a to carry out its resistance action on antibiotic compounds. The gyrA demonstrated unspecific reaction and binding on other genes such as gyrB, dnaN and pare as well as positive activation of the gyrB to carry out antibiotic resistance (Figure 6b). Tet (M) also demonstrated unspecific reaction and binding on a wide range of genes presented in Figure 6c to be involved in antibiotic resistance.

## Discussion

S. aureus is a part of the most clinically important pathogens causing severe economic losses worldwide [24]. The fast evolution of S. aureus resistant strains has rendered most current antibiotics ineffective, hence raised a global concern [24]. There is an urgent need for rapid detection of unique ARGs, which could aid in improvements in global surveillance [25, 26]. Therefore, we sought to identify antibiotic resistance genes from all available genomes of S. aureus strains. In this study, we identified a total of 32 ARGs across 11 S. aureus strain genomes. The expression of mepA and mepR in all the strains shows that these genes are found in a diverse range of resistant strains of S. aureus and it is highly conserved in their genome. We noticed that mepA and mepR are interrelated in agreement with the work of Kaatz et al. [27]. While the ARGs such as FosB3, vgaALC, those are unique to S. a. strain UTSW MRSA 55 and S. a. strain ISU935 respectively. This uniqueness was also observed in S. a. strain FDAARGOS_159 expressing mphC and SAT-4 (Figure 2). The S. aureus UTSW MRSA 55 is mostly resistant to Fosfomycin due to the expression of the FosB3 gene [28]. Research had shown that the FosB3 gene is expressed in a wide range of bacteria pathogens such as S. epidermidis, Enterococcus faecium and Bacillis subtilis and many others [29, 30].

The major mechanisms of antibiotic resistance identified in this work are the efflux pump complex or subunit conferring antibiotic resistance. This mechanism enhances efflux through overexpressed pumps is for bacterial pathogens such as S. aureus by which efficiently extrude antimicrobial drugs outside the cell [31, 32]. These transporters can extrude a wide range of unrelated compounds, which can lead to multidrug- resistant (MDR) [33]. This efflux of drugs that are shown by S. aureus was first discovered in Escherichia coli [33]. Some bacterial pathogens such as E. coli and other gram-negative bacteria also employed this mechanism to play other roles in pathogenicity of bacteria such as colonization, infection, and the persistence of microorganisms in the host. The antibiotic resistance gene cluster mechanism we found in S. aureus strains has been investigated in Vibrio cholerae O139 and O1 SXT by Hochhut et al. [34] and other bacterial pathogens.

The major methicillin resistance proteins identified and shared by majority of the strains are mecA, mecR1 and mecI. These genes have been well discussed as ARGs in most S. aureus strains [35] that include MRSA [36, 37]. The mecR1 ARG plays a role in the methicillin resistance by involving the penicillin-interactive as potential antirepressor. The mecI act as a transcriptional repressor that constitutively blocks the transcription of the gene for the penicillin-binding protein. The different mechanisms of how these genes are involved in the resistance to antibiotics have been well discussed by Lowy, [38]. The mecI and mecR1 ARGs regulate the mecA response to β-lactam antibiotics in a fashion similar to that of the regulation of blaZ by the genes blaR1 and blaI upon exposure to penicillin [36, 38]. These ARGs have been studied to lead β-lactam resistance [9, 37, 39]. Another gene that encodes a β-lactamase is the PC1 beta-lactamase (blaZ), which is one among the many MDR efflux pumps involved in biocide resistance [40]. S. aureus have been studied to have mechanisms for resistance to β-lactam antibiotics. The MRSA strains are also resistant to glycopeptide antibiotics such as vancomycin [36, 37, 38].

Our findings also identified other ARGs that are proposed to be associated with multidrug efflux pump in S. aureus efflux systems. Some of these ARGs are NorA, NorB, MepA. NorA and NorB are chromosomal genes that belong to the MFS and demonstrated some genetic diversity [33, 41]. The NorA and NorB is multidrug efflux pump, which belongs to the major facilitator transporter. NorA has a higher percent identity with other resistance genes in other bacterial pathogens such as Bmr from Bacillus subtilis and Tet (A) from Escherichia coli [42]. However, Bmr was not identified in the work. NorA is mostly expressed on the membrane that has an active efflux pump of a hydrophilic molecule such as quinolones [31]. NorB acts irrespective of NorA to carry out resistance against a wide range of quinolone compounds and other antibiotic agents [43]. The norB gene is also one of the best-studied multidrug efflux pumps that play a vital role in fluoroquinolone resistant in diverse strains S. aureus [33, 44]. Strains that express norB gene are resistant to wide range antibiotics such as norfloxacin, ciprofloxacin, and other structurally similar compounds as well as tetracycline at a lower level [44]. Our study shows that it has 14 TMHs, however, other researchers have shown that it is a 12 transmembrane segments protein. Detailed comparative studies had demonstrated that norB expressed identity with other genes such as Bmr and Blt of about 30% and 41% respectively of B. subtilis [33]. This also has identity of 39% with QacA of S. aureus [31]. The MgrA identified in the work had been known to regulate the expression of NorB (depending of the condition such as pH) in the different strains of S. aureus [31, 33].

One of the ARGs identified in this work to be unique to MRSA was the FosB3, which MRSA strains and other bacterial pathogens expressed to develop resistance to Fosfomycin antibiotics by chromosomal mutations [28]. FosB3 have some percent identity with other fosfomycin resistance genes such as FosA and FosC mostly expressed in Escherichia coli, Enterobacter cloacae and Klebsiella pneumoniae. S. aureus strains [28, 45]. These pathogens that express FosB3 gene have decreased ability in the L-α-glycerophosphate and hexose phosphate uptake system [28]. Hence, there is decreased affinity of fosfomycin resulting in resistance [28, 46]. Researchers have shown that overexpression of the target protein (MurA) for fosfomycin could also increase resistance [28]. The fosfomycin antibiotics mode of action is to inhibit the synthesis of the bacteria cell wall [28]. Therefore, fosfomycin have been employed in the treatment of bacterial infections that expressed multidrug-resistant (MDR) genes [47]. The identification of FosB3 in this study shows that fosfomycin antibiotics may not be infective in the treatment of S. aureus MRSA strains. Other bacterial pathogens where similar fosfomycin resistance genes can be found are Bacillus anthracis and Enterococcus faecium [28, 48].

Another group of other ARGs identified are the gene involved tetracycline resistance. These include the Tet (M), tet (K) and tet 38. The tet (M) had been shown to have inhibitory effects on tetracycline by a non-covalent. The Tet(K) that was identified in this study is a plasmid-encoded efflux pump that acts as a Na+(K+) / H+ antiporter and belongs to the MFS of transporters [49, 50]. However, results from these studies show that it has no TMHs. Studies have demonstrated that Tet (K) confers high levels of resistance to tetracycline, oxytetracycline and chlortetracycline [50]. However, S. aureus strains that have Tet (K) demonstrated lesser resistance to cycline-compound due to the lack of a hydroxyl substituent [31]. Therefore, S. aureus v521 and v605 strains studied in this work may be susceptible to antibiotics like minocycline, 6-demethyl-6-deoxytetracycline and doxycycline. This ARG is unable to pump out this substrate from the cytoplasm of the S. aureus [51]. The other tetracycline resistance gene Tet (38), which is also a chromosomally encoded drug efflux pump has an identity of 46% with the plasmid encoded Tet (K) of S. aureus. Our TMHs prediction shows that it comprise of 14 segments as an integral membrane protein. This is in agreement of other finding from other researchers [39, 50]. Although S. aureus strains studied in the work that expressed Tet (38) are more often resistant to tetracycline, however, they may be susceptible to other antibiotics such as minocycline and others [31]. Another important ARG identified in this study is LmrB, which had been known as proton-coupled multidrug antiporter also belonging to the MFS. Studies have shown that LmrB have an identity of about 39% to LmrS. Our study shows that it has 14 TMHs making it an integral protein to form a pore to extrude antibiotics such as lincomycin, kanamycin, linezolid, and fusidic acid. Hence strains of S. aureus possessing these ARGs are resistant to lincomycin and others [52].

The ArlS and ArlR genes identified in this work belong to the two-component regulatory system that regulates processes like adhesion, autolysis, multidrug resistance and virulence [31, 53]. These two genes are fully interconnected and regulate the expression of another AGR such as NorA, (which was also identified and discussed in this work) [54]. Their interconnection has been shown to negatively and positively regulate the Agr; virulence accessory gene, and SarA; staphylococcal accessory regulator, respectively. This regulation has been investigated to modulate several virulence factors such as serine protease, surface protein and alpha-hemolysin [55]. Research has shown that ArlS may probably act as a sensor protein at a histidine residue and transfers its phosphate group to ArlR [56].

One of the identified AGRs across the diverse strains studied and also in MRSA is the MepA gene. It is an efflux pump gene that belongs to the multidrug and toxin extrusion (MATE) transport protein family [33, 57]. Our finding shows that MepA have 12 TMHs and this is in accord with other researchers. The sav1866 ARGs was also identified in MRSA strains and is a multidrug transporter belonging to the ABC family. Our study shows that sav1866 have 5 TMHs. These TMHs could be part of the pore that triggers by ATP binding that is presumably the drug translocation pathway [49, 58]. The last ARG identified in this work that will be discussed is the gyrA, which is, involved in S. aureus antibiotic resistance to fluoroquinolones. S. aureus strains such as UCI 28, NCCP14562 and NCCP14558 were identified to express the gyrA, hence these strains will have resistance to fluoroquinolones antibiotics [59]. However, the strains could be susceptible to antibiotics such as Besifloxacin, tosufloxacin and structurally other similar compounds [45].

## Conclusion

We have identified about 32 genes that could have serious implication in antibiotic resistance. Phylogenetics analyses show the relationship of these ARGs. S. aureus are interconnected in function when one or more other genes are expressed. The strain with serious clinical implication on human and animal health is the S. aureus strain UTSW MRSA 55 expressing eight different ARGs. Seven of these genes are also expressed by other S. aureus strains. However, FosB3 is unique in MRSA strains. ARG are resistant to certain antibiotics. They are susceptible to several antibiotics in some strains. The results are helpful in S. aureus clinical surveillance in the context of antibiotic resistance.

## Figures and Tables

**Table 1:** Details on the different S. aureus strain genomes

S/N	*S. aureus* strain Genome	Accession No	No of base pair
1	*S. a.* strain UTSW MRSA 55	CP013231.1	28,98,306
2	*S. a.* strain 08-02300	CP015646.1	27,42,807
3	*S. a.* strain FDAARGOS_159	CP014064.1	28,01,188
4	*S. a.* strain ISU935	CP017090.1	28,61,508
5	*S. a.* strain NCCP14558	CP013953.1	29,55,147
6	*S. a.* strain NCCP14562	CP013955.1	29,10,941
7	*S. a.* strain V521	CP013957.1	30,85,555
8	*S. a.* strain V605	CP013959.1	30,89,367
9	*S. a.* subsp. aureus strain ISU926	CP017091.1	28,33,430
10	*S. a.* subsp. aureus strain UCI 28	CP018768.1	28,35,307
11	*S. a.* subsp. aureus USA300_TCH959 SCAFFOLD2	GG697986	10,18,247

**Table 2:** Number of Antibiotics Resistance Ontology genes shared/unique in 10 genomes

S/N	*S. a.* Strain Genome	No. (Montanaro et al.) shared/unique by Genome	ARO name
1	VD1, VD10, VD11, VD2, VD3, VD4, VD5, VD6, VD7, VD8 and VD9	2	mepA, mepR
2	VD10, VD11, VD2, VD3, VD4, VD5, VD6, VD7, VD8 and VD9	3	tet(38), norB, lmrB
3	VD1, VD10, VD2, VD3, VD4, VD5, VD6, VD7, VD8 and VD9	4	sav1866 arlR mgrA arlS
4	VD10, VD2, VD3, VD4, VD5, VD6, VD7, VD8 and VD9	5	antibiotic resistant fabI norA TaeA mfd dfrC
5	VD1, VD10, VD4, VD5, VD6, VD7, VD8 and VD9	1	mecA
6	VD10, VD4, VD5, VD6, VD7, VD8 and VD9	1	mecI
7	VD10, VD4, VD5, VD6, VD7 and VD8	1	mecR1
8	VD10, VD5, VD6, VD7, VD8 and VD9	2	ANT(9)-Ia ErmA
9	VD5, VD6, VD7, VD8 and VD9	1	tetM
10	VD4, VD7, VD8 and VD9	1	PC1 beta-lactamase (blaZ)
11	VD3, VD7 and VD8	1	APH(3')-IIIa
12	VD10, VD5 and VD6	2	ANT(4')-Ib Staphylococcus aureus gyrA conferring resistance to fluoroquinolones
13	VD7, VD8 and VD9	1	tet(K)
14	VD3 and VD4	1	bcrA
15	VD7 and VD8	2	dfrG AAC(6')-Ie-APH(2'')-Ia
16	VD3	2	mphC SAT-4
17	VD4	1	vgaALC
18	VD1	1	FosB3

Keys: ARO; Antibiotics Resistance Ortology, VD1; Staphylococcus aureus strain UTSW MRSA 55, VD2; S. a. strain 08-02300, VD3; S. a. strain FDAARGOS_159, VD4; S. a. strain ISU935, VD5; S. a. strain NCCP14558, VD6; S. a. strain NCCP14562, VD7; S. a. strain V521, VD8; S. a. strain V605, VD9; S. a. subsp. aureus strain ISU926, VD10;S. a. subsp. aureus strain UCI 28 and VD11;S. a. subsp. aureus USA300_TCH959 SCAFFOLD2			

## References

[1] Stevens DL (2005). Clinical Infectious Diseases..

[2] Belizário JE, Napolitano M. (2015). Frontiers in microbiology..

[3] Becattini S. (2014). Doctoral dissertation, ETH Zurich..

[4] Land M (2015). Functional & integrative genomics..

[5] Charkowski AO. (2016). Springer;.

[6] Thomer L (2016). Annual Review of Pathology Mechanisms of Disease..

[7] Montanaro L (2016). Frontiers in cellular and infection microbiology..

[8] Doudoulakakis A (2017). Journal of Clinical Microbiology..

[9] Humphreys H (2016). Journal of Hospital Infection..

[10] Bougnom BP, Piddock LJ. (2017). ACS Publications..

[11] Luepke KH (2017). Pharmacotherapy The Journal of Human Pharmacology and Drug Therapy..

[12] Laxminarayan R (2016). The Lancet..

[13] Landecker H. (2016). Body & Society..

[14] Vanderhaeghen W (2010). Epidemiology & Infection..

[15] Fair RJ, Tor Y. (2014). Perspectives in medicinal chemistry..

[16] Liu B, Pop M. (2008). Nucleic acids research..

[17] Sharma VK (2016). Chemosphere..

[18] Van Bambeke F (2008). Trends in pharmacological sciences..

[19] Everitt RG (2014). Nature communications..

[20] Jia B (2017). Nucleic acids research..

[21] Kumar S (2016). Molecular biology and evolution..

[22] Rambaut A. (2014). Institute of Evolutionary Biology, Univ. Edinburgh..

[23] Szklarczyk D (2017). Nucleic acids research..

[24] Bar-Gal GK (2015). Veterinary microbiology..

[25] Laxminarayan R (2013). The Lancet infectious diseases..

[26] Bradley P (2015). Nature communications..

[27] Kaatz GW (2006). Antimicrobial agents and chemotherapy..

[28] Fu Z (2016). PloS one..

[29] Xu X (2013). PloS one..

[30] Chen C (2014). Journal of medical microbiology..

[31] Andersen JL (2015). International journal of environmental research and public health..

[32] Astolfi A (2017). Journal of medicinal chemistry..

[33] Costa SS (2013). The open microbiology journal..

[34] Hochhut B (2001). Antimicrobial agents and chemotherapy..

[35] Ito T (2003). Drug Resistance Updates..

[36] Stegger á (2012). Clinical Microbiology and Infection..

[37] Monecke S (2013). Veterinary microbiology..

[38] Lowy FD. (2003). Journal of Clinical Investigation..

[39] Larsen J (2016). Antimicrobial agents and chemotherapy..

[40] Cabot G (2011). Antimicrobial agents and chemotherapy..

[41] Hooper DC, Jacoby GA. (2015). Annals of the New York Academy of Sciences..

[42] Roberts MC, Schwarz S. (2016). Journal of environmental quality..

[43] Aldred KJ (2014). Biochemistry..

[44] Truong-Bolduc Q (2005). Journal of bacteriology..

[45] Takahata S (2010). International journal of antimicrobial agents..

[46] Thirumal Kumar D (2017). Journal of Cellular Biochemistry..

[47] Modi SR (2013). Nature..

[48] Xu S (2017). Frontiers in microbiology..

[49] Li XZ, Nikaido H. (2009). Drugs..

[50] Ginn SL (2000). Journal of bacteriology..

[51] Guay GG, Rothstein DM. (1993). Antimicrobial agents and chemotherapy..

[52] Rodvold KA, McConeghy KW. (2014). Clinical infectious diseases..

[53] Fridman M (2013). Biochemistry..

[54] Sun J (2014). Biochemical and biophysical research communications..

[55] Mootz JM (2013). The University of Iowa.

[56] Sun F (2010). Journal of bacteriology..

[57] Kuroda T, Tsuchiya T. (2009). Biochimica et Biophysica Acta (BBA)-Proteins and Proteomics..

[58] Abdullah HQ (2017). Journal of Biological Chemistry..

[59] Ashley RE (2017). Biochemistry..

